# GWAS studies reveal a possible genetic link between cancer and suicide attempt

**DOI:** 10.1038/s41598-019-54812-9

**Published:** 2019-12-04

**Authors:** Konstantinos Voskarides, Andreas Chatzittofis

**Affiliations:** 1 Medical School, University of Cyprus, Nicosia, Cyprus; 2 Department of Clinical Sciences/Psychiatry, Umeå University, Umeå, Sweden

**Keywords:** Genetics, Cancer genetics

## Abstract

Inuit is the population with the highest incidence of suicide attempt and cancer in the world. Previous studies reported that people attempted suicide have a higher future risk for cancer. In view of these data, the largest available genome wide association studies (GWAS) for four major mental disorder groups were screened here for any common genes with all known cancer associated genes and oncogenes/tumor suppressor genes. A common genetic background came out only between suicide attempt and cancer (cancer associated genes analysis: RR = 1.64, p = 7.83 × 10^−5^; oncogenes/tumor suppressor genes analysis: RR = 2.55, p = 2.82 × 10^−22^), this supporting existing epidemiological data. Incidence/prevalence of both conditions was found to correlate with extreme cold geographical regions (adjusted R^2^ = 0.135, p = 3.00 × 10^−4^); this is not the case for other mental disorders. Our results show a possible genetic link between suicide attempt and cancer and a possible evolutionary connection of both diseases with extreme cold environments. These data are useful for future molecular studies or even for investigation of possible therapeutic protocols.

## Introduction

Suicide and cancer are currently among the leading causes of death and disease burden worldwide. In young people, suicide is the second cause of death and suicide rates seem to be on the rise. Similarly, cancer is the second leading cause of death globally with rates also increasing. Both diseases have a strong genetic background, more obvious in cancer but also in suicide^1,2^.

The relationship of cancer and suicide has been mainly focused on the effects of a newly cancer diagnosis leading to a subsequent increase in suicide risk. The relative risk for suicide after a cancer diagnosis is higher during the first year, in the presence of advanced cancer stage^3,4^ and mostly attributed to the stressful character of a cancer diagnosis^3^.

However, the relationship between cancer and suicide seems to be bidirectional. Lawrence *et al*. reported that people who attempted suicide had a higher future risk for cancer as well as higher cancer mortality^5^. This finding was replicated in a recent Swedish nationwide registry study^6^. This study was also the first to hypothesize a common molecular pathway between cancer and suicide through a dysregulation of the oxytocin system. Although the main hypothesis was rejected, this study replicated the finding of increased risk for future cancer in suicide attempters. Even though the increased cancer risk was mainly attributed to factors such as alcohol and tobacco use, the authors suggest that common biological pathways such as inflammation may also have an impact^6^. On the other hand, common confounders between cancer and suicide attempt, associated with lifestyle and personality, cannot be excluded as a possible cause of comorbidity.

Interestingly both suicide and cancer rates seem to follow a specific distribution in the world. Cancer has high incidence in areas of the world with extreme low temperatures possibly as an effect of evolutionary adaptation^7^ while there is positive relationship between suicide rates and geographic latitude^8^. Inuit people who have the highest cancer rates, and live in extreme low temperatures, also exhibit the highest suicide rates in the world, among indigenous or not indigenous populations^9,10^. Additionally, Alaska indigenous populations have the highest suicide rates in USA^11,12^. On the other hand, it is important to say that high suicide rate is observed in many other indigenous populations^10^. This shows that environmental factors and not only genetics contribute to the high suicide rates in those populations. Moreover, a recent study of human ancestry in Europe, proposed a genetic component related to adaptation to cold in order to explain the observed variation in suicide rates in different countries^13^.

To our knowledge there are no studies hitherto investigating possible common genetic pathways between suicide and cancer. Thus, the aim of this study was to investigate common genetic pathways between suicide and cancer as well as the relationship of suicide and extreme low temperature worldwide.

## Results

### Linear regression analysis between mental disorders and cancer

**Figure 1:**
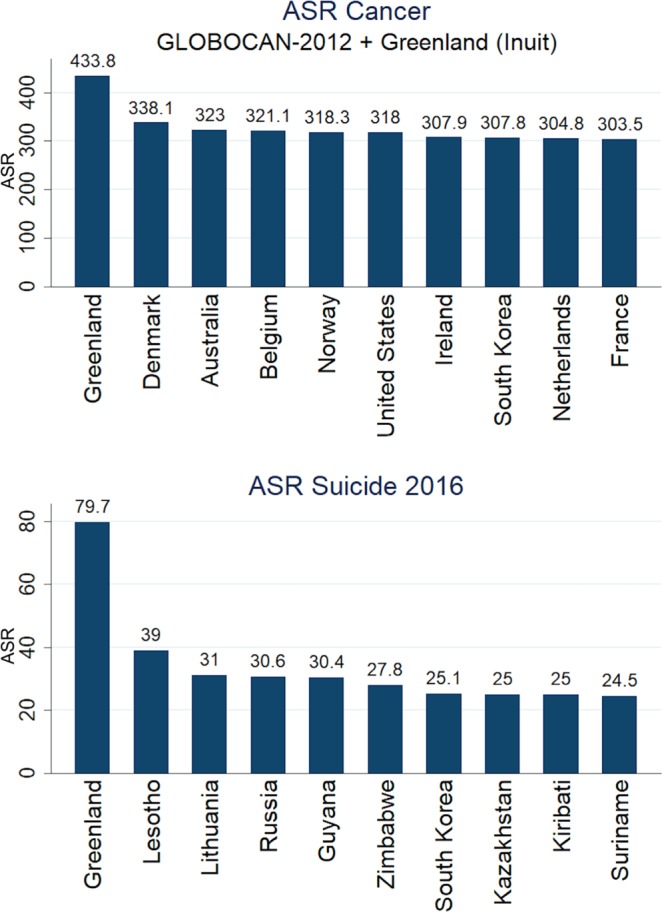
The 10 countries/populations ranking first in incidence for cancer and suicide deaths. Inuit rank first for both conditions (ASR: age-standardized rate). Inuit cancer ASR was adopted from^29^.

**Figure 2:**
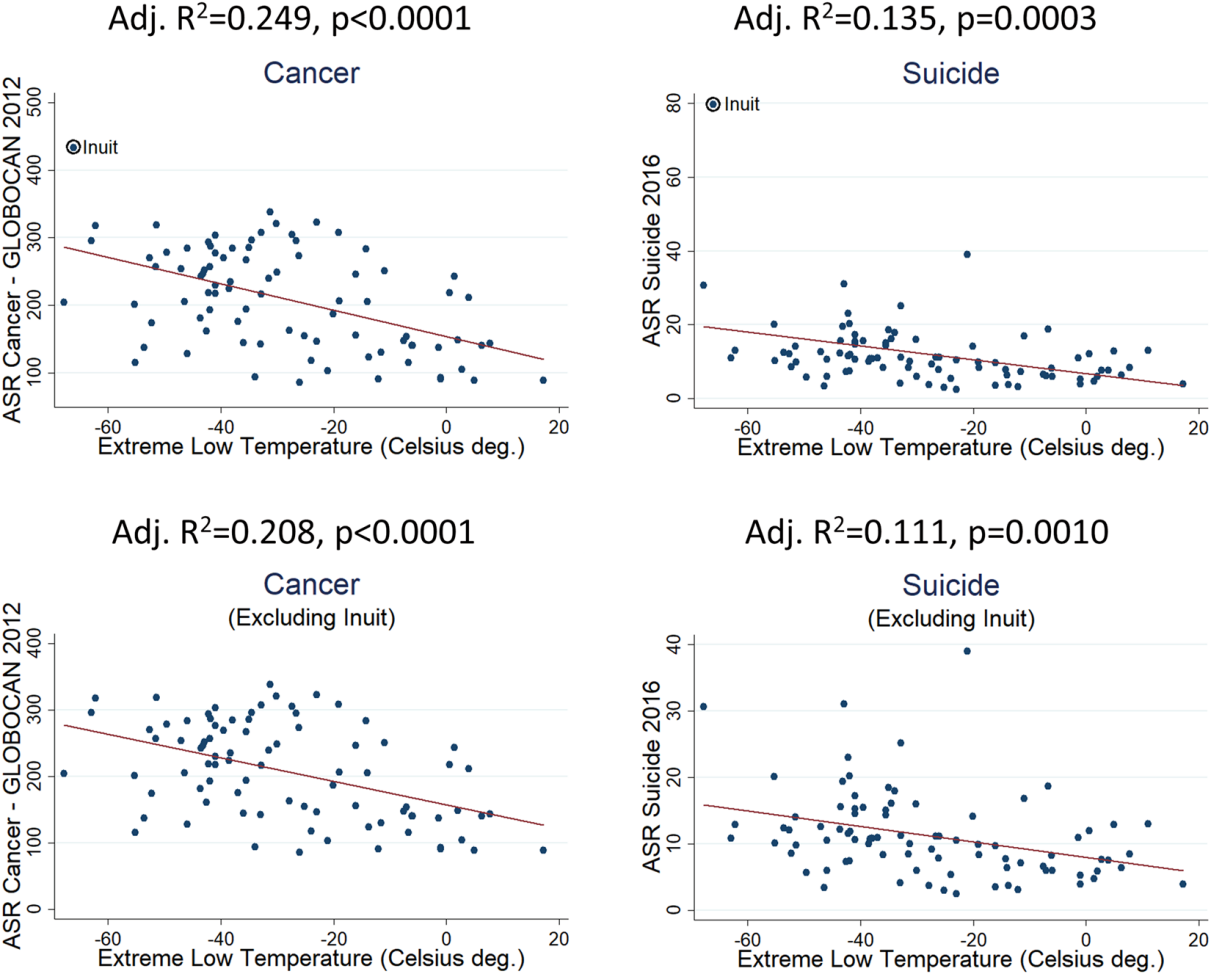
Linear regression of extreme low temperatures with incidence of cancer and suicide attempt (87 countries/populations). Positive linear association is observed for both disorders, by including or not including Inuit.

Interestingly, Inuit rank first worldwide in Cancer incidence and Suicide deaths (age adjusted, Fig. 1). A previous study^7^ found evidence that high cancer incidence (GLOBOCAN-2012 data) is linearly related with extreme low environmental temperatures, either for male or female. The same was found in the present study for Suicide deaths’ incidence (age adjusted data from “our world in data”, Fig. 2). Association is statistically significant by including or not including Inuit (Fig. 2). Significance is approximately the same by using the 2016 cancer prevalence rates from “our world in data” database or WHO 2016 suicide crude rates. Suicide association with extreme low temperatures exists for both male and female when analyzed separately (adjusted R^2^ = 0.177, p < 1.00 × 10^−4^; adjusted R^2^ = 0.074, p = 7.30 × 10^−3^, respectively), although significance is lower for female (WHO 2016 suicide crude rates, Supplementary Table 1).

**Figure 3:**
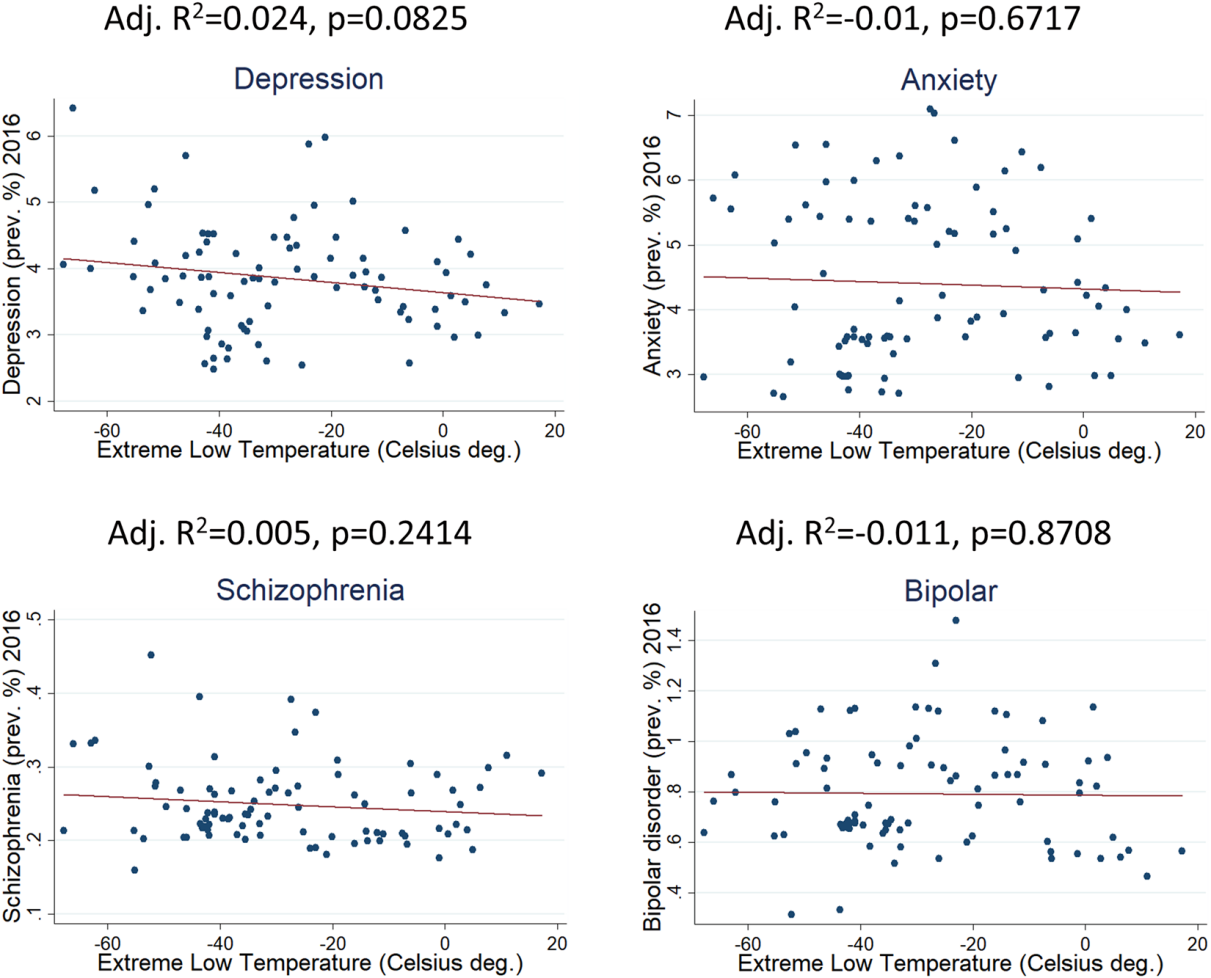
Linear regression of extreme low temperatures with incidence of four major mental disorders (87 countries/populations). No linearity is observed for any of those psychiatric conditions.

Not any similar association has been found for other mental disorders like Schizophrenia, Bipolar Disorder, Anxiety and Depression (Fig. 3).

### Cancer genes in common with mental disease associated genes

Cancer associated genes (CAG), Tumor suppressor genes (TSG) and Oncogenes (OG) were compared for similarities with genes that have been associated with mental disorders (Suicide, Depression, Anxiety, Schizophrenia). Our results show that 11% of the genes that have been found to be associated with suicide attempt are also genes that have been found to be associated with cancer (Table 1). This is far from being a result found by chance (RR = 1.64, p = 7.83 × 10^−5^). Even more, 1/5 (20%) of all genes that have been found to be associated with suicide attempt are genes that are considered as TSG or OG, this giving a high statistical significance (RR = 2.55, p = 2.82 × 10^−22^) (Table 1). The same analysis that has been performed for Depression, Anxiety, Schizophrenia, resulted to no statistical significance (Table 1). Analytical data are found in Supplementary Table 2.

**Table 1:** Mental disorder associated genes (MDAG) were statistically tested for containing various categories of cancer genes and housekeeping genes (HG) as a control test.

Population	x/MDAG	x/HGG	P-value	Fold enrichment	Mental disorder GWAS studies
**SUICIDE**					^1^
x: CAG	65/(590-65)	(1375-65)/(20479-590-1310)	**7.83 × 10**^**−5**^	1.641 (1.297, 2.075)	
x: TSG	66/(590-66)	(1037-66)/(20479-590-971)	**1.46 × 10**^**−9**^	2.209 (1.747, 2.794)	
x: OG	66/(590-66)	(724-66)/(20479-590-658)	**9.84 × 10**^**−17**^	3.164 (2.493, 4.016)	
x: TSG + OG	122/(590-122)	(1660-122)/(20479-590-1538)	**2.82 × 10**^**−22**^	2.551 (2.164, 3.008)	
x: HG	53/(590-53)	(3800-53)/(20479-590-3747)	**7.75 × 10**^**−11**^	0.484 (0.374, 0.627)	
**DEPRESSION**					^36,37^
x: CAG	20/(225-20)	(1375-20)/(20479-225-1355)	0.181	1.324 (0.869, 2.018)	
x: TSG	17/(225-17)	(1037-17)/(20479-225-1020)	0.092	1.492 (0.941, 2.366)	
x: OG	4/(225-4)	(724-4)/(20479-225-720)	0.200	0.503 (0.190, 1.332)	
x: TSG + OG	20/(225-20)	(1660-20)/(20479-225-1640)	0.623	1.097 (0.720, 1.670)	
x: HG	45/(225-45)	(3800-45)/(20479-225-3755)	0.547	1.078 (0.829, 1.402)	
**ANXIETY**					^34,35^
x: CAG	3/(31-3)	(1375-3)/(20479-31-1372)	0.462	1.441 (0.491, 4.230)	
x: TSG	3/(31-3)	(1037-3)/(20479-31-1034)	0.206	1.911 (0.651, 5.611)	
x: OG	0/(31-0)	(724-0)/(20479-31-724)	0.627	0.442 (0.028, 6.916)	
x: TSG + OG	3/(31-3)	(1660-3)/(20479-31-1657)	0.737	1.194 (0.407, 3.503)	
x: HG	7/(31-7)	(3800-7)/(20479-31-3793)	0.496	1.217 (0.634, 2.337)	
**SCHIZOPHRENIA**					^38^
x: CAG	4/(36-4)	(1375-4)/(20479-36-1371)	0.302	1.655 (0.656, 4.175)	
x: TSG	2/(36-2)	(1037-2)/(20479-36-1035)	0.704	1.097 (0.285, 4.224)	
x: OG	0/(36-0)	(724-0)/(20479-36-724)	0.639	0.382 (0.024, 5.999)	
x: TSG + OG	2/(36-2)	(1660-2)/(20479-36-1658)	0.766	0.685 (0.178, 2.638)	
x: HG	11/(36-11)	(3800-11)/(20479-36-3789)	0.083	1.647 (1.005, 2.697)	

Results show a highly significant enrichment of suicide associated genes with cancer genes (Fisher exact-test).

CAG: Cancer associated genes (1375 genes); TSG: Tumor suppressor genes (1037 genes); OG: Oncogenes (724 genes); HG: Housekeeping genes (3800 genes); HGG: Human genome genes (20479 genes); GWAS: Genome wide association studies.

### Housekeeping genes in common with mental disease associated genes

Since many cancer genes have a housekeeping role, there is a possibility that the significance that came out to be due to the enrichment of suicide association genes with housekeeping genes. Analysis showed that this is not the case since housekeeping genes are underrepresented in suicide association genes (Table 1). Regarding the other mental disorders’ association genes (Depression, Anxiety, Schizophrenia), housekeeping genes are found in expected frequencies (Table 1).

### Pathway analysis of suicide attempt/cancer common genes

All 169 cancer genes (CAG, TSG, OG) found in common with suicide associated genes were introduced in Pather v.14 for molecular pathway analysis. Seventeen molecular pathways came out with p-values (FDR) ranging from 9.07 × 10^−10^ to 5.28 × 10^−2^ (Supplementary Table 3). The five most significant pathways are listed in Table 2.

**Table 2:** Most significant pathways found in the 169 common genes between suicide associated genes and cancer genes (analysis by Panther v.14).

Pathway overrepresented (Panther v.14)	All human genes	Genes out of the 169	Fold enrichment	FDR
Cadherin signaling pathway (P00012)	157	15	11.66	9.07 × 10^−10^
CCKR signaling map (P06959)	173	12	8.47	2.13 × 10^−06^
FGF signaling pathway (P00021)	120	8	8.14	4.13 × 10^−04^
EGF receptor signaling pathway (P00018)	133	8	7.34	5.58 × 10^−04^
Angiogenesis (P00005)	172	9	6.39	5.84 × 10^−04^

FDR: False Discovery Rate.

## Discussion

Totally 1375 genes that have been associated with any type of cancer (see Methods) were compared with genes that have been discovered by polygenic risk score and trios analysis to be associated with suicide attempt^1^. A significant number of genes that predispose for suicide, are also associated with cancer (risk ratio = 1.64, p = 7.83 × 10^−5^) or they are oncogenes or tumor suppressor genes (risk ratio = 2.55, p = 2.82 × 10^−22^). Notably, nearly one fifth of all genes that have been associated with suicide attempt, are oncogenes or tumor suppressor genes. This is not the case for other mental disorders like depression, anxiety and schizophrenia (Table 1). For this analyses, only genetic data from the largest consortia studies were used, like the UK-biobank and the Psychiatric Genomics Consortium (Table 1). Interestingly, these results are in accordance with published epidemiological studies that showed that people who attempted suicide have a higher risk to get diagnosed with cancer in the future, when compared with the general population^5,6^. However, we would like to state here that most genes associated with suicide attempt came out by genetic statistical analysis and not through functional studies like oncogenes and tumor suppressor genes.

*In silico* analysis of common genes revealed possible genetic pathways in common between cancer and suicide, like the cadherin signaling pathway and the CCKR (gastrin/cholecystokinin receptor) signaling map. Some of these pathways have already been studied in suicide^14–19^. Since these results are coming from a genetic association, functional studies are needed to test further this evidence.

It is also very interesting that we found here that extreme low temperatures are related with suicide attempt rates. We believe that there are two possible explanations: (a) low temperatures may activate inflammation pathways in the brain, since such evidence already exists in animal models^20–22^ or (b) genetic variants that contributed to adaptation of humans to cold may also predispose for suicide attempt (no evidence exists yet). Further investigation is needed for clarifying the reasons of this association.

In conclusion, genetic association data (GWAS), environmental data (extreme temperatures), prevalence data (Inuit population) and clinical data, suggest a genetic link between suicide and cancer. It is important to notice that there does not seem to be a genetic relationship between other psychiatric diseases and cancer and these common genetic pathways are specific for suicide. Patients with suicidal behavior differ both clinically and biologically from non-suicidal patients^23–25^. Suicidal patients show increased inflammation with alterations in pro-inflammatory cytokines, have more often metabolic disorders and have more often dysregulation in stress adaptation^25,26^. These same factors are also contributing to carcinogenesis and tumor development^27^. Thus, it is possible that common genes in suicide and cancer mediate the biological vulnerability for both diseases. We believe that these results may be the beginning of a new research effort for identifying cancer patients at suicide risk and people that attempted suicide with high future cancer risk. This could be performed by investigating possible genetic or biochemical biomarkers. Early prevention of such conditions could save many lives from cancer or suicide attempt. Common genetic background of these two medical conditions may be the result of an ancient adaptation to special environmental conditions, like extreme low temperatures. Further studies on common genetic background and common molecular pathways of suicide attempt and cancer could contribute to future pharmacological targeting.

## Methods

### Data sources

Cancer incidence data (age-standardized Rates – ASR) for all countries were adapted from GLOBOCAN-2012 (http://globocan.iarc.fr)^28^. Cancer incidence for Inuit population was adapted by Young *et al*.^29^. Suicide incidence data (ASR) for 2016 were downloaded from “our world in data” database (https://ourworldindata.org/) and from World Health Organization (WHO) website (https://www.who.int/). Mental diseases’ age standardized prevalence data (2016) were downloaded from “our world in data” database (https://ourworldindata.org/).

Extreme Low Temperature data for 87 countries were retrieved from Arizona State University climate extremes archive (https://wmo.asu.edu/) and from reliable sources that are referred in List of weather records (https://en.wikipedia.org/wiki/List_of_weather_records).

All data above are listed in Supplementary Table 1. ASR is termed “new cases/time period/100,000 individuals. ASR data of GLOBOCAN-2012 are recorded for year 2012.

The methological approach that has been described above has been adopted by Voskarides, 2018^7^.

### Disease association genes and gene comparison

NHGRI-EBI GWAS catalog (https://www.ebi.ac.uk/gwas/)^30^ was used for downloading all genes that have been associated with cancer through GWAS studies (Supplementary Table 2) since May of 2017. NHGRI-EBI GWAS catalog is a continuously updating GWAS database. Keyword “cancer” and genetic association with P ≤ 5 × 10^−8^ were used as filtering criteria for retrieving all GWAS cancer association studies that are archived in NHGRI-EBI GWAS catalog. Totally 240 GWAS studies were included for analysis (Supplementary Table 2). All cancer association studies were downloaded except cervix cancer, due to its high correlation with infectious factors. GWAS for drug response and disease progression were excluded too. In order to create a “cancer associated genes” list (CAG), genes termed as “mapped” or “reported” by the NHGRI-EBI GWAS catalog, were included for listing. Genes in duplicate (same in different studies) were excluded in order to have only unique gene names. CAG list can be found to Supplementary Table 2. Non-protein coding genes “MIR” and “LINC” and genes of uncertain importance or existence (“LOC”) were not considered for the statistical analysis. Despite this, readers can find them listed in Supplementary Table 2.

A separate analysis was performed for protein coding oncogenes (OG) and tumor suppressor genes (TSG). Oncogenes list was downloaded from oncogene database^31^, http://ongene.bioinfo-minzhao.org/, and tumor suppressor genes were downloaded from tumor suppressor gene database^32^, https://bioinfo.uth.edu/TSGene/. All related analyses can be found in Supplementary Table 2.

A separate analysis was performed with housekeeping gene (HG). Housekeeping genes’ list was adapted by Eisenberg and Lavanon^33^. This list (3,800 genes) is based on analysis of next-generation sequencing (RNA-seq) data. As the authors state, at least one variant of these genes is expressed in all tissues uniformly. Analysis was performed by comparing this list for any common genes with genes under selection, under the same logic that it is described below. The housekeeping genes’ list and all comparisons performed can be found in Supplementary Table 2.

Genes associated with the three major mental disease groups (Depression, Anxiety, Schizophrenia) were adapted from GWAS studies that have been performed using the largest sample cohorts that exist since today^34–38^, like the UK-biobank and the Psychiatric Genomics Consortium (all cited in Table 1). Suicide associated genes were adapted from the study of Sokolowski *et al*.^1^, the only study that used trios genetic analysis and polygenic risk score analysis. We adopted all genes that were listed as significant in the GWAS studies we analyzed. These genes are all listed in Supplementary Table 2.

Every excel sheet of Supplementary Table 2 contains mental disease gene lists for comparison with cancer or housekeeping genes. Search for common genes was performed though the “duplicate” function of Microsoft Excel 2016 and genes in common are highlighted. The same approach has been used by Voskarides^7^.

Human genome genes were considered to be 20479^39^. Statistical approach is a 2 × 2 table: [(n1) cancer genes]/[(z) mental disease genes – (n1)] Vs [(n2) all cancer genes – (n1)]/[20479 – z – (n2-n1)]. The methological approach that has been described above is similar of this applied by Voskarides^7^.

### Statistical analysis

Please see previous section for a detailed description of the data analysis approach. All statistical analysis needed for this work was performed though the statistical package STATAv.14 (StataCorp LLC, Texas, USA). Basic statistical analysis included univariate linear regression, Fisher exact test for 2 × 2 tables and bar plots for incidence or prevalence presentation of various populations. Significant level alpha was set to 0.05.

Software Panther v.14[22] was applied for finding significant pathways overrepresented in the 169 common genes (Supplementary Table 3) found between suicide associated genes and cancer genes.

## Supplementary information

Supplementary Table 1

Supplementary Table 2

Supplementary Table 3
